# An Assessment of the Level of Protection Against Colibacillosis Conferred by Several Autogenous and/or Commercial Vaccination Programs in Conventional Pullets upon Experimental Challenge

**DOI:** 10.3390/vetsci7030080

**Published:** 2020-06-30

**Authors:** Dimitris Koutsianos, Hubert Gantelet, Giovanni Franzo, Mathilde Lecoupeur, Eric Thibault, Mattia Cecchinato, Konstantinos C. Koutoulis

**Affiliations:** 1 Department of Poultry Diseases, Faculty of Veterinary Medicine, University of Thessaly, 43100 Karditsa, Greece; dkoutsianos@yahoo.gr; 2 Ceva Biovac Campus, Research and Development Department, 49070 Beaucouzé, France; hubert.gantelet@ceva.com (H.G.); eric.thibault@ceva.com (E.T.); 3 Department of Animal Medicine, Production and Health (MAPS), University of Padua, 35020 Legnaro, Italy; giovanni.franzo@unipd.it (G.F.); mattia.cecchinato@unipd.it (M.C.); 4 Ceva Animal Health, Data Analysis Department, 33501 Libourne, France; mathilde.lecoupeur@ceva.com

**Keywords:** poultry, *Escherichia coli*, APEC, colibacillosis, autogenous vaccine, intratracheal challenge

## Abstract

The prevention of avian colibacillosis has historically been investigated through vaccination, with variable outcomes. Commercial live (attenuated) and inactivated vaccines are reported to have limited efficacy in the context of heterologous challenge. Autogenous vaccination, using field isolates, is widely used, but scarcely documented. Different vaccination programs, including a live commercial vaccine and/or an inactivated autogenous vaccine, were compared for three different avian pathogenic *Escherichia coli* (APEC) strain (serotypes O78, O18 and O111) challenges. On the pullet farm, four groups of conventional pullets received different vaccination protocols. Group A was kept unvaccinated (control group). Group B was vaccinated three times with a live commercial O78 *E. coli* vaccine (at one day old, 59 and 110 days of age). Group C was immunized twice (at 79 and 110 days) with a three-valence autogenous vaccine (O78, O18 and O111). Group D was vaccinated first with the commercial vaccine (at one day old and 59 days), then with the autogenous vaccine (110 days). Birds were transferred to the experimental facility at 121 days of age and were challenged 10 days later. In each group, 20 birds were challenged with one of the three APEC strains (O78, O18, O111); in total, 80 birds were challenged by the same strains (20 per group). The recorded outcomes were: mortality rate, macroscopic lesion score in target organs and the bacterial recovery of the challenge strain from bone marrow and pooled organs. When challenged with O78 or O111 strains, birds from groups C and D proved to be significantly better protected, in terms of lesion scoring and bacteriological isolation, than those of groups A and B. With the O18 challenge, only birds of group D presented a statistically significant reduction of their lesion score. To the authors’ knowledge, this is the first report on the efficacy of an immunization program in poultry that combines commercial and autogenous vaccines.

## 1. Introduction

*Escherichia coli* is a Gram-negative bacterium that typically inhabits the intestine of vertebrates and is considered a usual member of the digestive flora in animals. However, under favorable circumstances, it may acquire genes associated with pathogenicity/virulence and cause clinical disease in most domestic farmed species. Colibacillosis is a systemic fatal disease, which occurs when an *E. coli* strain escapes digestive barriers and invades the internal organs. It is reported as the most common infectious disease in poultry and is prevalent in laying production; its economic impact is substantial in the poultry industry [1,2] and potentially in public health [3]. In commercial layers, colisepticemia/polyserositis, peritonitis/oviduct infection/oophoritis, swollen head syndrome and colibacillosis of the lower respiratory system have been described in adult birds, while omphalitis is also observed in day-old chicks. The ability of such *E. coli* strains to cause localized and systemic disease is termed avian pathogenic *E. coli* (APEC). It has been tentatively related to certain serotypes, and is generally associated with a number of virulence factors [4,5,6]. The serotypes most frequently reported to cause colibacillosis in poultry are O1, O2 and O78 [7,8], while many other serotypes have been sporadically incriminated, such as O111 in layer chickens with colibacillosis and peritonitis [9,10]. Research projects in India and Greece also reported the presence of O111 among various *E. coli* serotypes in layers [11,12]. In Italy, investigations into colibacillosis outbreaks in laying hens identified 15 different serotypes, with O78, O2 and O129 being dominant in some cases [13], while O78, O88 and O2 were found most frequently by others [14]. In the United Kingdom, MacPeake et al. [15] found that the most prevalent serotype in layers was O78. A German study on colibacillosis in layers and broilers identified O2, O78 and O1 as the most prevalent serotypes [16]. Additionally, serotyping a collection of isolates obtained from different avian species from France, Spain and Belgium between 1992 and 2000 identified O1, O2, O5, O8, O18 and O78 as the most important serotypes [5]. Among APEC strains, multiple serogroups, as well as varied virulence factors, are implicated in colibacillosis, with several alternative pathways seeming to lead to pathogenicity.

APEC strains can infect chickens (*Gallus gallus*) through different routes; however, respiratory (inhalation of contaminated dust) and oral routes are considered the main infection pathways in conventional laying production [17]. The extent to which cloacal ascending infection, leading to salpingitis, influences intra-flock transmission among laying hens is not well understood, but is documented [18]. Under experimental conditions, respiratory (inhalation of contaminated aerosol and intratracheal inoculation) and intravaginal routes have been clearly demonstrated as efficient transmission pathways [19]. The intratracheal inoculation route has been proposed as a standard model, which is closer to mimicking the natural contamination of production birds [20,21,22]. Several factors may impact on the receptivity of the birds’ lower respiratory tracts to APEC infection, such as viral (infectious bronchitis virus) [21] or bacterial (*Mycoplasma spp*.) concurrent infections or environmental stressors (high ammonia levels) [23,24].

Colibacillosis control has historically been performed through the curative and preventative use of different classes of antimicrobials. However, increased antimicrobial resistance and a judicious use of antibiotics now requires alternative control strategies to protect animal and public health [25]. The implementation of such strategies started much earlier in laying hen production, because the regulations on maximum residue levels in eggs for human consumption limited the products available to poultry practitioners to a handful. The immunization of pullets/hens against colibacillosis with live, inactivated or sub-unit vaccines has been reported, with variable outcomes [6]. Live vaccines have been repeatedly proven to contribute positively to bird protection [26,27,28,29,30], although exceptions have also been reported [31]. A commercial live vaccine is presently available in most regions; it was registered in the EU in 2013 [32] and it is an attenuated O78 *E. coli* strain and provides effective protection upon challenge with O78 wild strains [33]. However, this type of vaccine is less efficient against heterologous strains (i.e., belonging to other serogroups) [23]. Inactivated vaccines can also be effective against colibacillosis [34,35]. Autogenous vaccines are produced from pathogenic strains isolated from within the affected flock where the vaccine will be used. They are widely used in the field for colibacillosis control [36,37]. By nature, each autogenous vaccine is different, and reports on the impact of their usage are sporadic. As far as colibacillosis in layers/breeders is concerned, two reports have been published, with a negative [38] and a positive outcome [37]. However, to the authors’ knowledge, no publication has reported so far on the use of autogenous vaccines in a colibacillosis vaccination program, together with a commercial vaccine, evaluated by intratracheal challenge. The aims of the present study were multiple: i. to assess the efficacy of a colibacillosis autogenous vaccine in conventional pullets upon challenge after the start of the laying period, ii. to compare the preventative efficacy of this autogenous vaccine to that of a commercial live vaccine and iii. to evaluate the combination of a live commercial vaccine and an autogenous vaccine in an original immunization protocol.

## 2. Materials and Methods

### 2.1. Origin of Vaccinated Pullets

Pullets were reared on a conventional Greek farm with recurrent problems of colibacillosis: increased mortality, macroscopic lesions of omphalitis, pericarditis and perihepatitis at necropsy. Samples collected during the previous rearing cycles had repeatedly confirmed the presence of O78 and O111 strains of *E. coli* in the lesions. The farm consisted of two houses, each conducted an all-in all-out policy, but not in synchrony. The trial protocol was shared with the famer and his staff before the date scheduled for the reception of a new batch of day-old chicks. The 4000 conventional day-old chicks (ISA Brown) were divided into four groups (A, B, C, D) of 1000 each in the hatchery. Day one vaccination was performed at the hatchery against Marek disease (HVT + Rispens, Nobilis^®^ Rismavac + CA126, MSD, Kenilworth, NJ, USA), Newcastle disease virus (Hitchner B1, Cevac^®^ UNI L, Ceva Animal Health, Libourne, France) and Infectious Bronchitis virus (Massachusetts B48 strain, Cevac^®^ Mass L, Ceva Animal Health). The four groups of chicks were placed in different rows inside the same house and were all vaccinated against Newcastle disease, IBV, infectious bursal disease, avian rhinotracheitis, *Salmonella* and infectious laryngotracheitis. These birds were sampled after hatching and were resampled at the end of the rearing period for the serology (ELISA, Biochek, Reeuwijk, The Netherlans) of *Mycoplasma gallisepticum* and *Mycoplasma synoviae.* All these tests provided negative results. These birds remained in the same house until transfer at 121 days of age, the usual age to transfer future laying hens to the egg production facility. Additionally, each group was subjected to a different vaccination protocol against APEC:

Group A was left unvaccinated;

Group B was vaccinated (spray) with a live commercial vaccine against O78 (Poulvac^®^ *E. coli*, Zoetis), on days 1, 59 and 110;

Group C was vaccinated with an autogenous vaccine (intramuscular injection in the musculus pectoralis) on days 79 and 110. The autogenous vaccine contained inactivated valences against O18, O78 and O111 and had been prepared in Good Manufacturing Practices GMP-compliant Biovac laboratories (Ceva Animal Health, Beaucouzé, France);

Group D was vaccinated (spray) with a live commercial vaccine against O78 (Poulvac^®^ *E. coli*, Zoetis), on days 1 and 59, and then with the autogenous vaccine (intramuscular route) on day 110.

### 2.2. Transfer to Experimental Laying Facility

The experimental procedure was approved by the Animal Ethics Committee (46/5.12.2017) of the University of Thessaly. At 121 days old, 80 pullets were randomly selected from group A and 60 birds from groups B, C and D and were transferred to the experimental units (Code: ΕL-41ΒΙO/exp-05) of the Department of Poultry Diseases, Faculty of Veterinary Medicine, University of Thessaly, Karditsa, Greece. They were placed in rooms containing four pens each, each pen containing 20 birds at a density of nine birds/m^2^, in compliance with European Union’s Council Directive 74/EC/1999 [39]. In three rooms, each pen contained birds from the same group and each group (A, B, C, D) was represented. In a fourth room, only one pen was occupied, by 20 pullets from group A (negative control).

The birds were reared in their pens for 10 days, with feed and water ad libitum, in order to acclimatize them to their new environment. During this period, light duration was increased by 1 h per week, reaching 16 h at 131 days old.

### 2.3. Experimental Inoculation

Challenge strains: three different *E. coli* strains were used for the challenge trial. An O78 strain (ref. 170564) and an O111 strain (ref. 170655) that had recently been isolated from lesions of pericarditis and perihepatitis, respectively, in pullets from a previous batch on the same farm were used. Upon consultation with Ceva Biovac, France, on the design of the autogenous vaccine, it was agreed to include a third valence with a historical O18 strain (ref.50176). The characterization of APEC of the three strains was done according to the predictive model of virulence published by Schouler et al. [5] (Table 1).

The pathogenicity of each of these strains had been assessed through experimental inoculation in a previous experiment [40]. Briefly, groups of unvaccinated 105-day-old conventional pullets were intratracheally challenged with one of the above *E. coli* strains (0.5 mL of a concentration approximately 10^9^ CFU/mL), in compliance with Antao et al. [22] and Landman et al. [19]. Pathogenicity was evaluated by recording mortality and scoring macroscopic lesions at necropsy (heart, liver, air sacs, lungs, spleen); each strain was found to be pathogenic.

Preparation of the inoculum: strains were cultivated overnight in sheep blood agar (Bioprepare, Greece). A single colony from each culture was then suspended in 50 mL of TSB broth and incubated for 24 h at 37 °C The bacterial concentration was estimated through decimal dilutions of the TSB suspension, spread on TSA agar, incubated for 24 h at 37 °C and then counted. The bacterial concentrations (CFU/mL) used for challenge were 9.8 × 10^8^, 1.1 × 10^9^ and 1.6 × 10^9^ for the O78, O111 and O18 strains, respectively.

Challenge: at 131 days of age, which corresponds to the start of the laying period in conventional hens, each bird in the first three rooms was individually inoculated with 0.5 mL of the appropriate bacterial suspension, with a curved canula, via the intratracheal route. Room #1 was challenged with the O18 strain, room #2 with the O78 strain and room #3 with the O111 strain. The 20 birds in the 4th room were challenged with saline, as a negative control.

Evaluation of pathogenicity: the birds were monitored on a daily basis for clinical signs. Mortality was recorded daily, and all dead birds were removed from pens and necropsied. Macroscopic lesions were evaluated according the scoring system described by Antao et al. [22], which includes lesions on the heart, liver, spleen, thoracic air sacs and lungs. Peritonitis lesions were also scored (Table 2).

Seven days after the experimental challenge, all the surviving birds were humanely euthanized and immediately necropsied. Macroscopic lesions were evaluated in the same way. During necropsy, samples were collected from all birds (*n* = 260), to attempt to cultivate *E. coli* from the femoral bone marrow and from a pool of internal organs (heart, liver, spleen and air sacs).

All the samples were cultured on MacConkey agar (Bioprepare, Greece) and sheep blood agar (Bioprepare, Greece). Suspect colonies and isolates were sent to Ceva Biovac’s Laboratory for identification confirmation and serotyping, respectively. Three primary outcomes were considered: mortality rate, total macroscopic lesion score and isolating the challenge strain from dead/euthanized birds.

### 2.4. Statistical Analysis

Difference in lesion scores were assessed using the Kruskal–Wallis test. When significant differences were observed, a pairwise Mann–Whitney post hoc test with Bonferroni correction was performed. A total lesion score was calculated for each bird, being the sum of all organ scores.

Differences in the number of deaths between groups was evaluated by Fisher’s exact test. A similar approach was used to compare the frequency of challenge strain re-isolation. The level of significance was set to *p* < 0.05 for all considered methods.

## 3. Results

### 3.1. Clinical Signs

Depression, ruffled feathers, nasal exudates, coughing and gasping appeared in the days following the challenge in birds from groups A and B, independent of the challenge strain. Birds in groups C and D did not show any clinical signs, apart from a pronounced reduction in water and feed intake over the hours following the experimental challenge. Birds from the negative control group maintained their natural appetite and behavior over the seven-day observation period. The results are detailed for the three primary outcomes.

### 3.2. Mortality

The seven-day mortality is shown in Table 3. In the O78 challenge, dead birds were observed only in groups A and B, with a statistically significant higher mortality rate (*p*-value = 1.966094 × 10^−5^) than in groups C and D. No significant differences between groups were found in the O18 and O111 challenges (*p*-value = 1 and 0.24, respectively).

### 3.3. Mean Organ Lesion Scores

The mean lesion scores (per organ and total) are detailed for each group and challenge trial in Table 3.

In the O18 challenge, group D presented a significantly lower heart mean lesion score (*p*-value = 0.01672) compared to group A. The liver and lung mean lesion scores were not significantly different between these groups (*p*-value = 0.1574 and 0.1304, respectively). The air sac mean lesion score from Group D was also significantly lower than that of any other group (*p*-value = 0.0001195). The spleen mean lesion score was also significantly lower in group D than in groups A and C (*p*-value = 0.002427). A comparable significant result was observed for the peritonitis mean lesion score between group D and groups B and C (*p*-value = 0.0102). Overall, groups A, B and C presented a comparable, elevated mean total lesion score, while that of group D was significantly lower (*p*-value = 0.001858), even when the splenic lesion score was subtracted (*p*-value = 0.002819).

In the O78 challenge, groups C and D presented significantly lower mean lesion scores for heart (*p*-value = 3.987 × 10^−7^), liver (*p*-value = 2.534 × 10^−9^) and peritonitis (*p*-value = 2.823 × 10^−6^), as well as a lower total mean lesion score (*p*-value = 6.477 × 10^−8^). The splenic mean lesion score was not statistically different between groups (*p*-value = 0.3916). In groups C and D, the mean air sac lesion score was significantly lower than in group B (*p*-value = 0.002458). The mean lung lesion score was significantly lower in group C as compared to group A (*p*-value = 0.02745).

In the O111 challenge, groups C and D presented significantly lower mean lesion scores for heart (*p*-value = 7.33 × 10^−7^), liver (*p*-value = 4.357 × 10^−6^), spleen (*p*-value = 0.0001912), air sacs (*p*-value = 7.576 × 10^−7^), peritonitis (*p*-value = 9.929 × 10^−9^) and total mean score (*p*-value = 3.293 × 10^−9^) than groups A and B. The mean lung lesion score was not statistically different between groups.

### 3.4. Re-isolation of the Challenge Strains

The results of the bacteriological isolation of the challenge *E. coli* strains from bone marrow and pooled organs in the corresponding groups are detailed in Table 4.

In the O18 challenge, there was no statistically significant difference for the frequency of strain recovery between groups from neither bone marrow (*p*-value = 0.43) nor pooled organs (*p*-value = 0.0853). However, the lowest number of isolations was achieved in group D.

In the O78 challenge, groups C and D presented a significantly lower number of birds allowing the re-isolation of the challenge strain from bone marrow (*p*-value = 4.357553 × 10^−7^) and pooled organs (*p*-value = 1.59604 × 10^−10^) than in groups A and B. When the isolation results are considered at the individual bird level (positive for either sample), the same significant difference was observed.

In the O111 challenge, re-isolation failed from both organs for all birds in groups C and D (bone marrow: *p*-value = 0.05; pooled organs: *p*-value = 5.9358 × 10^−7^), compared to groups A and B. No significant difference was observed between groups A and B, as far as the re-isolation of the challenge strain was concerned.

## 4. Discussion

This experimental evaluation of different pullet APEC vaccination programs demonstrates through three primary outcomes (mortality, mean lesion score and challenge strain re-isolation) that autogenous vaccination provides a significant improvement in the protection level, either alone or in combination with a live commercial vaccine. To the authors’ knowledge, this work is the first to explore such a combined use of avian colibacillosis immunization methods.

The experimental model used in the present experiments builds on previous pathogenicity trials that have established both inoculum concentration and the route of infection. The intratracheal route was chosen because it mimics the natural way birds are infected in the field. It has been standardized for APEC inoculation by Antao et al. [22], who used different concentrations of bacterial suspension, ranging from 10^7^ to 10^9^ CFU/mL. Additionally, Landman et al. [19] aimed at reproducing *E. coli* peritonitis by challenge through different routes, including the intratracheal route, with inoculum quantities ranging from 10^5^ to 10^9^ CFU/hen. In previous pathogenicity trials, our team confirmed, with O78 and O111 strains isolated from the same pullet-rearing farm and with the O18 reference strain, that the inoculation of 0.5 mL of a bacterial suspension at approximately 10^9^ CFU/mL via the intratracheal route is a valid model for reproducing avian colibacillosis [40]. Under experimental conditions, the spectrum of protection conferred by immunization has been explored by different authors and is generally considered to reach almost full protection in homologous challenge conditions and rarely so with heterologous challenge [31,37].

Although autogenous vaccine use is widespread in the poultry industry, especially in birds with a long production life (breeders, laying hens, turkeys, etc.), there are a limited number of publications reporting on their efficacy. Landman and Van Eck [37] reported on the protection provided by an autogenous vaccine prepared from an APEC strain isolated from the bone marrow of birds with egg peritonitis syndrome. The homologous experimental challenge demonstrated that pullets were almost fully protected, while the heterogenous challenge provided inconclusive results. The present experiment relates to both homologous (groups C and D) and heterologous (group B) challenge conditions. In groups C and D, the autogenous vaccine was prepared from inactivated APEC strains. In group B, although the live commercial vaccine contained an O78 strain, it may have differed from the field O78 strain present on the farm, and it was not expected to provide protection against the O111 strain also circulating on that farm [6], since the product’s specification did not include non-O78 APEC strains [32]. This is further substantiated by the O18 experimental challenge on group B, where even though no mortality was induced in unvaccinated birds (that strain had proved pathogenic in the same model on another occasion, Koutsianos et al., [40]) and other authors mention field cases of colibacillosis linked to O18 strains (Blanco et al., [41]; Dou et al., [42]), limited protection was observed; mortality, mean total lesion score and challenge strain re-isolation were not significantly different from those in non-vaccinated birds (group A). Nevertheless, the extent of heterologous protection needs further exploration, since group D (mixed vaccination program) showed milder clinical signs, a lower re-isolation rate and a significantly lower mean lesion score than all other groups (A–C) upon O18 challenge. The combination of live and autogenous vaccines seems to have triggered a wider immunization spectrum, as observed in turkeys by Sadeyen et al. [43]. In commercial broilers, the immune response produced by a spray live vaccine against *E. coli* is mainly a cellular response, especially relevant to the sites in contact with the pathogen, as indicated by data obtained by Filho et al. [44].

In the O78 challenge, the pathogenic effect of the strain on the birds was immediate and profound. This is in line with field [10,45] as well as experimental challenges (aerosol inoculation) in *Gallus gallus* [46]. There was no significant difference between groups A and B in any of three primary outcomes, substantiating the limited protection provided by the live commercial vaccine against the present field O78 strain. On the other hand, birds in groups C and D proved significantly protected against that strain, for these same criteria. The significantly lower recovery of the challenge strain suggests that the immune protection elicited by the vaccination programs used in groups C and D effectively stopped the systemic diffusion of the APEC strain in birds. Vaez Zadah et al. [47] reported that a booster dose of an O2 outer membrane protein (OMP)-based vaccine induces a significant increase in specific antibody titers. In contrast, Li et al. [38] reported the limited clinical efficacy of an O78 autogenous vaccine even though they measured a significant rise in antibody titers but used different vaccination (subcutaneous) and inoculation (intra-oviduct) routes in broiler breeders. Most publications, however, report reduced mortality and macroscopic lesions as the common outcome of *E. coli* O78 vaccination, regardless of the vaccine used and experimental inoculation route [27,28,29,30].

The O111 challenge confirmed the pathogenicity of that strain (group A), although with a more limited impact on both mortality and lesions than that of the O78 strain. This has been reported from field cases [9,10,11]. The pathogenicity of an O111 strain was also reproduced through the intramuscular inoculation of both specific-pathogen-free (SPF) and conventional pullets by Zanella et al. [9]; the oronasal route proved less efficient under these experimental conditions. In the present work, groups C and D evidenced a significantly improved protection in terms of mean lesion score, and group D in terms of mean lesion score and challenge strain re-isolation. A comparison with the group B results allows us to infer that the improved protection may be ascribed to the use of the autogenous vaccine.

Different virulence factors and different combinations thereof can be present in different APEC strains [16,23], while some virulence factors are also present in non-pathogenic *E. coli* strains [17]. That may partly explain why wide heterologous protection is difficult to achieve. Until an immunogenic protein common to APEC strains is identified, autogenous vaccines may fill the gap left by the limited spectrum of the commercially available vaccines. On the other hand, the combination of both vaccine types in a common immunization program seems to be a promising tool to limit the welfare, health and economic impacts of colibacillosis and the antimicrobial footprint of the laying industry.

## 5. Conclusions

Several immunization programs against colibacillosis in pullets were assessed against experimental challenges. Two applications of an autogenous vaccine consisting of O78, O111 and O18 strains and two applications of a live commercial vaccine combined with a single application of the autogenous vaccine provided a significant positive effect against intratracheal challenge with either the O78 or O111 strain. Furthermore, the results of the O18 challenge point to the idea of combining both vaccine types in order to expand heterologous protection since only the group that had been vaccinated with both vaccine types presented a statistically significant reduction in mean lesions. Further investigation should be undertaken to better characterize this observation. In the context of limiting antimicrobial usage in the poultry industry and, given the importance of avian colibacillosis in terms of animal welfare and potential public health impact, an effective and multi-serotype vaccination program is a long-awaited strategy. The combination of commercial and autogenous vaccines seems a promising preventive management tool.

## Figures and Tables

**Table 1:** Virulence genes identified and the genetic pattern for *E. coli* strains used for the autogenous vaccine production.

Ref BIOVAC	*E. coli* Strain *	iutA	A12	D7	A9	PAP	FEL	papG1	papG2	Genetic Pattern
**170564**	O78	+	+	-	-	+	+	-	+	A
**170655**	O111	+	+	+	-	-	+	-	-	B
**50176**	O18	+	+	+	+	-	-	-	-	B

* The three strains used for the autogenous vaccine production.

**Table 2:** Lesion scoring systems used for each target organ. Lesions were scored according to Antao et al. [22], except for peritonitis, the scoring of which was based on the authors’ observations.

Target Organ	Score	Description
Air sacs	(0–3)	0: absence of lesions 1: presence of a small amount of fibrin in the pericardium and/or a minor change in the transparency and thickness of pericardium 2: presence of a medium amount of fibrin in the pericardium and/or an intermediate change in the transparency and thickness of pericardium 3: presence of a massive amount of fibrin in the pericardium and/or an important change in the transparency and thickness of pericardium
**Liver**	(0–2)	0: normal liver appearance 1: small presence of fibrine material on the liver surface, the changing of natural liver color and texture 2: increased amount of fibrin covering the liver surface
**Spleen**	(0–1)	0: normal size and appearance of spleen 1: enlarged and congested spleen
**Heart**	(0–3)	0: normal transparent pericardium 1: pericardium is vascular, lack of transparency 2: increased pericardiac fluid that is (or is not) transparent 3: severe fibrinous pericarditis
**Lungs**	(0–5)	0: absence of lesions 1: presence of a limited affected area with necrosis/fibrin covering 1/5 of lungs 2: One or more lesions covering 2/5 of lungs 3: lesions covering 1/2 of lungs 4: lesions covering 4/5 of lungs 5: lesions covering 5/5 of lungs
**Peritonitis**	(0–2)	0: absence of lesions 1: presence of spots of fibrin inside the peritoneum 2: presence of mass fibrinous material inside the peritoneum

**Table 3:** Mortality, total and mean scoring for macroscopic lesions per organ in the O78, O18 and O111 challenge trials. Macroscopic lesion scores are expressed for each organ by mean ± standard deviation.

Challenge Strain	O78				O18				O111			
Vaccination protocol	A	B	C	D	A	B	C	D	A	B	C	D
Mortality (%)	9/20^a^ (45%)	7/20^a^ (35%)	0/20^b^ (0%)	0/20^b^ (0%)	0/20 (0%)	1/20 (5%)	1/20 (5%)	0/20 (0%)	2/20 (10%)	0/20 (0%)	0/20 (0%)	0/20 (0%)
Heart	2^a^ ± 0.72	2^a^ ± 0.85	1^b^ ± 0.45	1.05^b^ ± 0.51	1.5^a^ ± 0.76	1.65^ab^ ± 1.03	1.55^ab^ ± 0.99	0.8^b^ ± 1.00	1.6^a^ ± 0.99	1.55^a^ ± 0.51	0.55^b^ ± 0.51	0.55^b^ ± 0.51
Liver	1.3^a^ ± 0.80	1.1^a^ ± 0.78	0^b^ ± 0.00	0.1^b^ ± 0.44	0.55 ± 0.82	0.9 ± 0.91	0.9 ± 1.02	0.35 ± 0.67	0.95^a^ ± 0.82	0.6^a^ ± 0.68	0^b^ ± 0.00	0.1^b^ ± 0.30
Spleen	1 ± 0.00	0.95 ± 0.22	1 ± 0.00	1 ± 0.00	0.95^a^ ± 0.22	0.8^ab^ ± 0.41	0.9^a^ ± 0.30	0.5^b^ ± 0.51	1^a^ ± 0.00	1^a^ ± 0.00	0.6^b^ ± 0.50	0.6^b^ ± 0.50
Air sacs	1.75^ab^ ± 0.78	2^a^ ± 0.72	1.2^b^ ± 0.61	1.25^b^ ± 0.85	1.75^a^ ± 0.78	1.55^a^ ± 0.75	1.6^a^ ± 0.94	0.55^b^ ± 0.75	1.4^a^ ± 0.59	1.6^a^ ± 0.50	0.75^b^ ± 0.55	0.6^b^ ± 0.50
Lungs	2.15^a^ ± 1.42	1.6^ab^ ± 1.23	1^b^ ± 0.79	1.3^ab^ ± 0.80	1.6 ± 0.75	1.2 ± 0.89	1.1 ± 1.02	1.1 ± 1.16	1.3 ± 1.38	0.7 ± 0.73	0.9 ± 0.85	0.65 ± 0.74
Peritonitis	1.1^a^ ± 0.55	1^a^ ± 0.64	0.2^b^ ± 041	0.4^b^ ± 0.50	0.6^ab^ ± 0.82	0.95^a^ ± 0.88	0.95^a^ ± 0.88	0.2^b^ ± 0.52	0.95^a^ ± 0.68	0.8^a^ ± 0.52	0^b^ ± 0.00	0.1^b^ ± 0.30
Total	9.3^a^ ± 2.57	8.65^a^ ± 3.24	4.4^b^ ± 1.35	5.1^b^ ± 2.04	6.95^a^ ± 2.99	7.05^a^ ± 3.96	7^a^ ± 4.02	3.5^b^ ± 3.00	7.2^a^ ± 2.74	6.25^a^ ± 1.80	2.8^b^ ± 1.36	2.6^b^ ± 1.72
Total w/o spleen*	8.3^a^ ± 2.57	7.7^a^ ± 3.14	3.4^b^ ± 1.35	4.1^b^ ± 2.04	6^a^ ± 2.97	6.25^a^ ± 3.72	6.1^a^ ± 4.87	3^b^ ± 2.86	6.2^a^ ± 2.74	5.25^a^ ± 1.80	2.2^b^ ± 1.28	2^b^ ± 1.41

* The lesion score of spleen has been subtracted. No results are reported for the non-vaccinated non-challenged group (negative control) due to a total lack of mortality and macroscopic lesion scoring for each euthanized bird. a,b: values without superscript do not differ significantly (*p* > 0.05), while different superscripts in the same row correspond to statistically significant differences between groups (*p* < 0.05).

**Table 4:** Bacteriological isolation of the challenge *E. coli* strains from bone marrow and pooled internal organs in the corresponding groups.

Vaccination Protocol	Nature of Sample											
	Bone Marrow				Pooled Organs				Birds*			
	*Challenge strain*				*Challenge strain*				*Challenge strain*			
	O78	O18	O111	Total	O78	O18	O111	Total	O78	O18	O111	Total
**A: No vaccination**	11^a^/20	3/20	4^a^/20	18/60	15^a^/20	8/20	12^a^/20	35/60	16^a^/20	9/20	12^a^/20	37/60
**B: Live O78 vaccine (×3)**	13^a^/20	5/20	1^ab^/20	19/60	15^a^/20	8/20	4^ab^/20	27/60	16^a^/20	10/20	4^ab^/20	30/60
**C: Autogenous vaccine (×2)**	0^b^/20	4/20	0^b^/20	4/60	1^b^/20	9/20	0^b^/20	10/60	1^b^/20	9/20	0^b^/20	10/60
**D: Live O78 (×2) + autogenous vaccine (×1)**	2^b^/20	1/20	0^b^/20	3/60	4^b^/20	3/20	0^b^/20	7/60	4^b^/20	3/20	0^b^/20	7/60

* A bird is considered positive when re-isolation is achieved from bone marrow, from pooled organs or both. No results are reported for the non-vaccinated non-challenged group (negative control) because *E. coli* strain isolation was not found in any euthanized bird. a,b: values without superscript in the same column do not differ significantly (*p* > 0.05), while different superscripts correspond to statistically significant differences between groups (*p*-value < 0.05).
